# Red Blood Cells: Chasing Interactions

**DOI:** 10.3389/fphys.2019.00945

**Published:** 2019-07-31

**Authors:** Virginia Pretini, Mischa H. Koenen, Lars Kaestner, Marcel H. A. M. Fens, Raymond M. Schiffelers, Marije Bartels, Richard Van Wijk

**Affiliations:** ^1^Department of Clinical Chemistry and Haematology, University Medical Center Utrecht, Utrecht University, Utrecht, Netherlands; ^2^Theoretical Medicine and Biosciences, Saarland University, Homburg, Germany; ^3^Department of Laboratory of Translational Immunology and Department of Pediatric Immunology, Wilhelmina Children’s Hospital, University Medical Centre Utrecht, Utrecht, Netherlands; ^4^Experimental Physics, Saarland University, Saarbrücken, Germany; ^5^Department of Pharmaceutics, Utrecht Institute of Pharmaceutical Sciences (UIPS), Faculty of Science, Utrecht University, Utrecht, Netherlands; ^6^Paediatric Haematology Department, Wilhelmina Children’s Hospital, University Medical Centre Utrecht, Utrecht, Netherlands

**Keywords:** red blood cells, interactions, membrane proteins, phospholipids, plasma proteins, platelets, endothelial cells, pathogens

## Abstract

Human red blood cells (RBC) are highly differentiated cells that have lost all organelles and most intracellular machineries during their maturation process. RBC are fundamental for the nearly all basic physiologic dynamics and they are key cells in the body’s respiratory system by being responsible for the oxygen transport to all cells and tissues, and delivery of carbon dioxide to the lungs. With their flexible structure RBC are capable to deform in order to travel through all blood vessels including very small capillaries. Throughout their in average 120 days lifespan, human RBC travel in the bloodstream and come in contact with a broad range of different cell types. In fact, RBC are able to interact and communicate with endothelial cells (ECs), platelets, macrophages, and bacteria. Additionally, they are involved in the maintenance of thrombosis and hemostasis and play an important role in the immune response against pathogens. To clarify the mechanisms of interaction of RBC and these other cells both in health and disease as well as to highlight the role of important key players, we focused our interest on RBC membrane components such as ion channels, proteins, and phospholipids.

## Introduction

Red blood cells (RBC) are the most abundant cell type in human blood. They are devoid of nuclei, ribosomes, mitochondria, and other organelles, which are important in other cell types to perform specific functions critical to cell survival (Adams, 2010). This unconventional cell composition has evolved in order to allow accumulation of hemoglobin, a protein that is responsible for the delivery of oxygen (O_2_) to peripheral tissues. In a typical healthy adult, every second 2 million of newly formed RBC enter the circulation from the bone marrow and at the same time about the same number is cleared (Higgins, 2015). RBC production, or erythropoiesis, is a tightly regulated process in which new RBC are continuosly produced in the bone marrow niche, sitting side by side in a rich environment with different cells and other tissues like endothelial cells (ECs), osteoblasts, stromal cells, hematopoietic cells as well as extracellular matrix proteins. In the bone marrow they are in direct contact with cell adhesion molecules, growth factors and cytokines (Dzierzak and Philipsen, 2013). During the last step of the RBC maturation process, which takes place during the first couple of days in the bloodstream, the reticulocytes or premature RBC, enter the peripheral blood. They go through a selective sorting process in which they lose 20% of the plasma membrane and the remaining RNA content. The RBC membrane in particular undergoes different morphological and structural changes from the maturation stage until the clearance stage. They undergo multiple and often tightly regulated processes, in order to remodel their structure starting with the loss of the complex organelles system and the consequent acquisition of the typical biconcave shape.

This process effectuates a selection, segregation and depletion of membrane proteins (Moras et al., 2017), like the decline of Na^+^/K^+^ATPase, the sodium-hydrogen antiporter 1 (NHE1), Glycophorin A (GPA), cluster of differentiation 47 (CD47) and cluster of differentiation 36 (CD36), Duffy antigen/chemokine receptor (DARC) and Kell antigen (XK) system with the loss of Transferrin Receptor (CD71) and intercellular adhesion molecule-4 (ICAM4). In contrast, other relevant membrane proteins are increased during maturation of RBC, when compared to reticulocytes, such as band 3, Glycophorin C (GPC), rhesus protein (Rh), Rh-associated glycoproteins (RhAG), XK, and GPA (Minetti et al., 2018).

After maturation RBC acquire the remarkable ability of being deformable in response to external forces (Huisjes et al., 2018) and use this in order to pass through the narrowest blood capillaries (Viallat and Abkarian, 2014). The importance of this characteristic becomes more evident when defects and abnormalities related to RBC shape and/or deformability lead to drastic and premature cell clearance. These changes can provide key information in establishing a differential diagnosis and categorizing different diseases (Ford, 2013).

**FIGURE 1:**
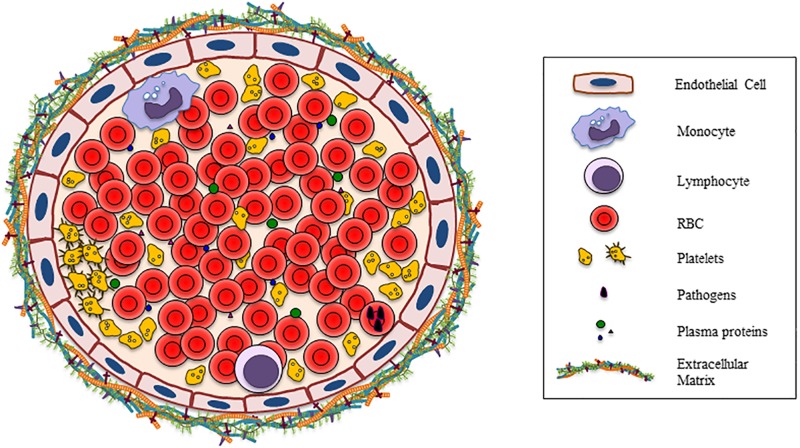
Vessel section shows all the possible cells, plasma proteins, bacteria, and the extracellular matrix that are involved in the interaction with RBC.

Much has been reported on the complexity of the interactions between the different components of the mature RBC membrane and other cells in the last decades. However, a complete overview of these interactions is lacking. In this review, we focus on the broad and diverse types of interactions that have been described to occur between RBC and other cells present in peripheral blood, and the consequences of these interactions. Many of the interactions known to occur are mediated by RBC membrane components (Figure 1).

## Structure of RBC Membrane and Membrane Skeleton

In RBC, the cytoskeleton and the plasma membrane are extremely and closely connected to create a fundamental and complex structure called membrane skeleton. This is essential for the shape and reversible deformability of RBC. Thanks to the membrane structural integrity maintenance; RBC are flexible and able to survive in the circulation (Lux, 2016). RBC can deform with linear extension up to an estimated 250%, whereas a 3–4% increase in surface area results in lysis of the cell. RBC owe these unique membrane properties due to interaction of the plasma membrane envelope with the cytoskeleton. The plasma membrane is composed of a lipid bilayer with embedded transmembrane proteins that form multi-protein complexes. The bilayer itself consists of equal proportions of cholesterol and phospholipids (Cooper, 2000). For structural integrity, the bilayer links to the membrane skeleton through two macroprotein complexes: the ankyrin complex and the junctional complex (also known as the 4.1R complex). The RBC skeleton is a protein meshwork in which the most important components are spectrin, actin, actin-associated proteins, protein 4.1R and ankyrin. The membrane skeleton constitutes of spectrin tetramers that bind short actin filaments that in turn form a pseudohexagonal arrangement with six triangular spectrins binding one actin filament. Each arrangement has three junction complexes and three ankyrin complexes that facilitate membrane-cytoskeleton linkages (Goodman and Shiffer, 1983; Mankelow et al., 2012; Lux, 2016). The ankyrin complex can link ankyrin to β-spectrin on one side and band 3 and RhAG in the RBC membrane, on the other side. The junction complex links membrane proteins GPC and GPD, XK, Rh and Duffy onto the actin-spectrin cytoskeleton through interaction with protein 4.1R (Mohandas and Gallagher, 2008; Burton and Bruce, 2011; Lux, 2016).

**FIGURE 2:**
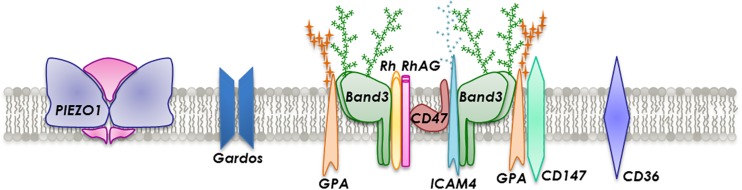
Section of the RBC membrane with a focus on the composition of integral membrane proteins incorporated into a phospholipid bilayer.

There are more than 50 types of transmembrane proteins embedded in the lipid bilayer that are involved in transport, adhesion and structural integrity (Figure 2). Transmembrane transport is executed by several proteins such as band 3, aquaporin-1, glucose transporter 1 (GLUT1), Kidd antigen protein, RhAG and various ion transporters, dependent on the cargo. Proteins involved in adhesion or cell-cell interactions include ICAM-4 and Lu. Generally, RBC are not considered adhesive cells but several studies have reported the expression of a large number of adhesion molecules (Altankov and Serafimov-Dimitrov, 1990; de Oliveira and Saldanha, 2010; Weisel and Litvinov, 2019). However, in a number of pathological and disease-associated circumstances, such as in sickle cell disease (SCD), malaria, polycythemia vera, hereditary spherocytosis, retinal vein occlusion and diabetes mellitus, RBC notably change their behavior and become stimulated and consequently adhesive to each other (Steffen et al., 2011) and in particular to the endothelium (Coste et al., 2001; Kaul, 2008; Grossin et al., 2009b; Colin et al., 2014).

## Transmembrane Transport-Proteins Mediated Interactions

### Ion Co-transporters

A large part of the RBC membrane is occupied by anion exchanger proteins. These allow RBC to maintain the correct balance between extracellular and intracellular water and solute content, in order to preserve the regular physiological functionality and homeostasis of RBC. For example, in case sodium influx exceeds potassium efflux the RBC swell, and conversely, if the outflow of potassium exceeds sodium influx into the RBC, the cells shrink (Gallagher, 2017). For this reason, a drastic change or a defect in the mechanism controlling the hydration balance could evolve into altered and unusual behavior of RBC (Azouzi et al., 2018).

### Band 3

The major anion exchanger protein in RBC membranes is band 3. This transmembrane glycoprotein (100 kDa) provides integrity to the RBC membrane. In addition, the N-terminal cytoplasmic side is a key site for the connection to the membrane skeleton, glycolytic enzymes and deoxyhemoglobin, whereas the C-terminal integral membrane side includes the anion-exchange transporter role and supports carbon dioxide transport (Lux, 2016). Changes at the hamoglobin level can be transmitted to the membrane and this causes oxidative stress and band 3 oligomerization which plays an important role in the macrophages-mediated clearance of altered and old RBC by forming senescence-induced-antigens that are recognized by natural antibodies (NAbs; Klei et al., 2017; Azouzi et al., 2018).

The numerous binding sites connect band 3 with other membrane proteins. Hereby, a crucial network is provided allowing the transduction of signals from the membrane to the cytoskeleton, and vice versa, thereby regulating the flexibility, stability, and deformability of RBC. Rifkind and Nagababu showed that the interaction of hemoglobin (Hb) with band 3 under hypoxic conditions is critical for generation of RBC membrane changes that trigger the removal from the circulation (Rifkind and Nagababu, 2013). In patients with diabetes mellitus, band 3 is glycated (AGE) and binds to the receptor for advance glycation end products (RAGE) present on ECs and this enhances the oxidant stress in the vessel wall (Schmidt et al., 1996; Grossin et al., 2009a). The protein glycation induces a series of changes which is associated with high risk of vascular complications, which is particularly apperent in diabetes mellitus in the microcirculation of the eyes leading to retinopathy and diabetic nephropathy (Wautier et al., 2004).

### PIEZO1

The recently discovered non-selective cation channel PIEZO1, is a mechanosensor that plays an important role in maintaining RBC volume homeostasis. It is known that genetic mutations of PIEZO1 are the primary cause of hereditary xerocytosis. These genetic alterations influence channel kinetics, response to osmotic stress and membrane trafficking (Glogowska et al., 2017), thereby causing a decrease in total cellular cation, calcium and potassium content without proportional intake of sodium and water, ultimately leading to significant dehydration (Bae et al., 2013; Honoré et al., 2015; Patel et al., 2015). The nature of this channel is primarily and directly dependent on the activation by mechanical forces (i.e., poking, stretching, and shear stress) (Cahalan et al., 2015; Gottlieb, 2017; Parpaite and Coste, 2017). There are multiple hypotheses regarding PIEZO1 activation mechanisms. One sustains the “force-from-lipids” model, in which membrane tension can cause lipid reorganizations around the protein causing the channel to open. Another theory is the “force-from-filaments,” which supports a model of interaction and tethering of the channel with extracellular matrix or intercellular cytoskeletal proteins (Murthy et al., 2017). Functional studies have shown that specific regions of the protein are more susceptible to mechanical perturbation than other regions. Moreover, there are many mechanical stimuli, such as shear stress, generated from the fluid flow over cells, that could interact and consequently activate PIEZO1 channels (Murthy et al., 2017). However, these mechanisms are still not completely understood and PIEZO1 is recognized as a potential candidate for the stretch-induced cation pathway and is involved in RBC aging, and circulatory shear stress (Bagriantsev et al., 2014). It has also been described to play a role in malaria parasite (*Plasmodium falciparum*) invasion (Zuccala et al., 2016; Ma et al., 2018). Interestingly, it was found that a third of the African population carry the novel variant of PIEZO1 that is associated with malaria resistance *in vitro* (Ma et al., 2018).

### KCNN4-Gardos Channel

The Gardos channel, or KCNN4/IK-1, is a calcium-activated potassium channel which is present in a low copy number on the RBC membrane. In fact the estimated number of channels per RBC measured is around 10 (Grygorczyk et al., 1984; Brugnara et al., 1993; Thomas et al., 2011; Kaestner, 2015). Gardos channel-mediated interactions with other cell types are indirect and often mediated by two other membrane proteins: PIEZO1 and an other unknown receptor. An example is the ability of RBC to change their ratio shape/volume to pass through narrow capillaries and interstices (Danielczok et al., 2017). The mechanism behind this is the activation of PIEZO1 resulting in increased intracellular Ca^2+^ which in turn initiates Gardos channel activity. This also implicates that Gardos channels play a role in disorders related to the RBC hydration like in hereditary xerocytosis (Gallagher, 2017; Rapetti-Mauss et al., 2017).

Regarding the interaction between the Gardos channel and a putative associated unknown receptor on the RBC membrane, a link was found between the endothelin receptor and Gardos activity with elevated levels of cytokines such as endothelin-1, interleukin-8, and platelet activator factor (PAF) in plasma of SCD patients: this disease is characterized by the intrinsic property of hemoglobin S to sickle under deoxygenation. Sickling is enhanced under various conditions, including dehydration due to activation of Gardos channels with consequently loss of K^+^ (Rivera et al., 2002). Moreover, SCD RBC have been shown to interact with vascular ECs, thereby stimulating the release of endothelin-1 and regulating the expression of the corresponding gene in culture. This mechanism could contribute to the vaso-occlusive events seen in SCD (Phelan et al., 1995).

Recently, pathological alterations were discovered correlating with mutations in the Gardos channel gene (Fermo et al., 2017): in fact, in some cases, patients with hemolytic anemia have been reported carrying exclusively these mutations responsible for this disease (Glogowska et al., 2015; Gallagher, 2017). These mutations changes the Ca^2+^ sensitivity affecting the activation threshold but also modifies functional properties making the channel more active leading to dehydrated RBC with a deficit in intracellular potassium (Archer et al., 2014; Andolfo et al., 2015; Rapetti-Mauss et al., 2015; Fermo et al., 2017).

### Other Transport-Proteins

Other important RBC transport-proteins are GLUT-1, responsible for glucose trafficking, ABCB6 (adenosine triphosphate-binding cassette), linked to heme biosynthesis and porphyrin transport, urea passive transporter (Azouzi et al., 2013), to preserve the osmotic stability and deformability of the cell (Macey, 1984), aquaporin-1, key pore for water transport and fundamental for the metabolism and transport of CO_2_, and volume-regulated anion channels (VRAC), a small conductand, stretch-activated channel, with the essential and recently descovered component SWELL1 (LRRC8A), located in proximity of the channel pore and responsible fort he regulation of cell volume homeostasis (Qiu et al., 2014; Syeda et al., 2016; Gallagher, 2017; Hsu et al., 2017). In addition, there are also regulatory proteins that cooperate with transport channel functionality like stomatin, which is a major protein of human RBC membranes that mainly interacts with the channels mentioned above (Rungaldier et al., 2013). It is currently unknown if these transport-proteins can induce interactions with other cells.

## Phospholipids Mediated Interactions

**FIGURE 3:**
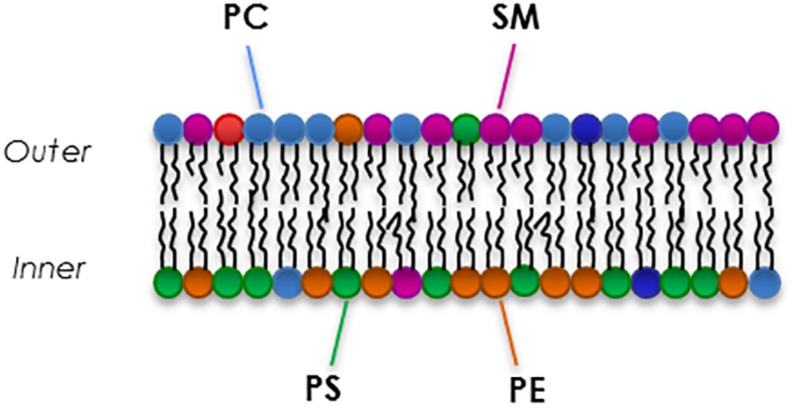
An example of the composition of the major phospholipids of the RBC membrane.

Red blood cells membranes are composed of a complex mixture of different kinds of phospholipid species that differ in head group and side chains (Kuypers, 2008). The lipid bilayer composition is similar to any other cell: there is an equal distribution of cholesterol and phospholipids (Cooper, 2000), while there is an asymmetrical proportion of the four major phospholipids between the two leaflets (Figure 3). In fact, the outer leaflet of the membrane is rich in phosphatidylcholine (PC, 27% of total membrane phospholipids) and sphingomyelin (SM, 23%), while the inner leaflet is mostly constituted by phosphatidylethanolamine (PE, 30%) phosphatidylserine (PS, 15%) and the minor phosphoinositide (PI, 5%) (Goodman and Shiffer, 1983; Pasvol et al., 1992; Kuypers and de Jong, 2004; Mohandas and Gallagher, 2008; Fujimoto and Parmryd, 2016). The translocase proteins present in the RBC membrane, flippase, floppase, and scramblase are responsible for the movement of phospholipids. Flippase and floppase maintain and regulate the asymmetry in response to different stimuli and signals (Smith and Lambert, 2003; Hankins et al., 2015). In contrast, activation of scramblase is known to be involved in loss and disruption of the membrane phosholipids asymmetry that is essential for maintaining lipid homeostasis in RBC membranes (Pretorius et al., 2016).

The unbalance proportion and composition of the membrane lipids drives to defects that are able to influence membrane protein activation and in some cases leading to diseases such as hemolytic anemia: in fact, an increased phosphatidylcholine in the RBC have been reported in some intermediate syndromes of hereditary xerocytosis, associated with PIEZO1 defect (Clark et al., 1993; Imashuku et al., 2016; Gallagher, 2017).

### Phosphatidylserine (PS)

Phosphatidylserine is an amino-phospholipid that is known to play a crucial role in mediating the recognition of senescent RBC, serving as an eat-me signal. During aging, upon injury of cells, or under certain pathologic conditions, scramblase translocates PS from the inner leaflet to the outer, leading to increased concentration on the external surface (Nguyen et al., 2011). It was shown that RBC of SCD patients expose PS upon deoxygenation and this process is mediated by Ca^2+^ influx activating the Gardos channels. In fact, scramblase requires Ca^2+^ for scrambling PS (Weiss et al., 2011; Wesseling et al., 2016). PS exposure on RBC has a second fundamental physiological function which is promoting coagulation. Generally, exposure of PS is described to increase the adhesiveness (Kaestner et al., 2012; Kaestner and Minetti, 2017). Regarding its role in coagulation, it was shown that RBC can directly bind to CD36 and PS-receptor (PSR) on ECs (Closse et al., 1999; Manodori et al., 2000; Setty et al., 2002; Heppel, 2008), as well as through CXCL16 or CD36 present on platelets after their activation (by ADP or thrombin) (Walker et al., 2013). PSR gets upregulated after activation of microvascular ECs by lipopolysaccharides (LPS), cytokines, hypoxia, and heme (Setty and Betal, 2008). CXC chemokine ligand 16 (CXCL16) is a direct ligand for PS exposed on RBC (Borst et al., 2012). CXCL16 gets strongly upregulated when stimulated with inflammatory cytokines such as IFN-γ or TNF-α (Abel et al., 2004), but also by peptidoglycans of the bacterial wall (Abed et al., 2013). Another way PS-exposure is promoted, is upon platelet interaction with FasR on RBC through FasL (Mackman, 2018). Moreover, key interaction induced by PS is the initiation of cell clearance, which involves different phagocytic cells such as macrophages, and usually takes place in liver and spleen. Various receptors have been identified to mediate direct binding via recognition of PS such as Tim1, Tim4, and Stabilin-1, leading to phagocytosis (Kobayashi et al., 2007; Park et al., 2009). Furthermore, there are several bridging molecules, or opsonins, like lactadherin, thrombospondin, Gas6, Protein S, that can facilitate macrophages recognition upon interaction with PS expose on RBC (de Back et al., 2014). Lastly, it was shown that changes in the cholesterol/phospholipid ratio in RBC had a marked effects on the PS exposure: in fact, an excess of cholesterol inhibites the PS exposure, whereas cholesterol depletion increases it (van Zwieten et al., 2012) and this could lead to increased susceptibility to the clearance of these cells.

## Glycoproteins Mediated Interactions

### Glycophorins

The glycophorin family is a group of transmembrane proteins [glycoproteins A-D (GPA-D)] that play an important role in regulating mechanical properties of RBC (Chasis and Mohandas, 1992). They comprise the most abundant integral type of proteins of the RBC membrane with around a million copies per cell (Alenghat and Golan, 2013; Aoki, 2017).

Glycophorin A is the major syaloglycoprotein responsible for the net negative surface charge of the cell membrane: for this reason the heavily glycosylated glycans on the extracellular domain are important to minimize the cell-cell interactions and RBC aggregation (Chasis and Mohandas, 1992; Poole, 2000). With this complex extracellular structure, GPA has a role into the composition of the pericellular matrix, the glycocalyx (Poole, 2000). GPA is also involved in pathogen recognition, acting as a decoy receptor in RBC. It mediates the binding of pathogens to the surface of RBC thereby preventing the invasion of these organisms into important tissues (Morera and MacKenzie, 2011). Consequently, pathogen load is reduced because pathogens bound to RBC are commonly cleared by macrophages in the spleen (Baum et al., 2002).

Additionally, it is shown that the sialoglycoproteins (GPA, GPB, and GPC) play a crucial role in the invasion of RBC by malaria parasites (i.e., *P. falciparum*). RBC deficient in any of the sialoglycophorins resist infection by merozoites to varying degrees (Pasvol, 1984). For instance, Gerbich-negative blood group (Ge-), characterized by defective GPC receptor expression, has been associated in malaria resistance with high prevalence in malaria-endemic regions (Williams, 2006).

### Intercellular Adhesion Molecule 4 (ICAM-4)

Intercellular adhesion molecule 4 is a glycoprotein, which plays a crucial role in cell-cell interaction or adhesion with potential significance in a variety of physiological processes including hemostasis and thrombosis (de Oliveira and Saldanha, 2010). It binds to different members of integrin receptor families expressed on white blood cells (WBC) and ECs (Goel, 2002; Hermand et al., 2002) and can get activated in RBC under the influence of the hormone epinephrine. Upon exposure to epinephrine, ICAM-4 is able to bind directly to α_v_β_3_ integrin on ECs (Zennadi et al., 2004; Kaul et al., 2006; Trinh-Trang-Tan et al., 2010; Zhang et al., 2017) through activation of the cAMP-PKA pathway. Epinephrine stimulates the β2-adrenergic receptors on RBC, which in turn stimulate adenylyl cyclase. Adenylyl cyclase catalyzes the production of cAMP from ATP. Finally, ATP activates protein kinase A (PKA) through the extracellular signal-regulated kinase 1/2 (ERK 1/2) cascade and phosphorylates ICAM-4 to an active state that can bind to α_v_β_3_ integrin on activated ECs. In this process, A-kinase anchoring proteins (AKAPs) are critical since they guide PKA to specific locations in the cell, initiating phosphorylation of neighboring RBC receptors (Zennadi et al., 2012; Maciaszek et al., 2013; Zhang et al., 2017). It has also been shown that ICAM-4 has a potential physiological significance in mediating RBC-platelet interactions in hemostasis and thrombosis (Hermand et al., 2004). The platelet integrin α_IIb_β_3_ (fibrinogen receptor GPIIb-IIIa) has been identified as a receptor for ICAM-4 *in vitro* (Hermand et al., 2002) and under flow conditions (Du et al., 2014). A specific ICAM-4 binding peptide could competitively block binding of fibrinogen to integrin α_IIb_β_3_. Blocking the interaction via ICAM-4 and integrin α_IIb_β_3_ reduced thrombin and fibrin deposition, with thicker and less fiber branches being present in the formed thrombi. This is likely to be of clinical consequence, since a significantly higher tail bleeding time was seen in mice in which the ICAM4-integrin α_IIb_β_3_-pathway was blocked. The mechanism behind decreased thrombus formation could be that the ICAM-4-α_IIb_β_3_ interaction causes intracellular signaling in platelets activating other platelets (Du et al., 2014). By blocking α_IIb_β_3_, the binding of RBC to platelets decreased from 72 to 29% (Du et al., 2014) indicating that α_IIb_β_3_ is involved in RBC-platelet interaction. In addition, it has been suggested that this interaction leading to RBC-platelets aggregates might contribute to vaso-occlusive events typically seen in SCD (Hermand et al., 2004).

Finally, other studies on adhesion of hemopoietic and non-hemopoietic cells, reported that ICAM-4 might also interact with α_v_β_1_ and α_v_β_5_ integrins (Hermand et al., 2004).

### Cluster of Differentiation 36 (CD36)

Cluster of differentiation 36 is a highly glycosylated protein capable of binding to thrombospondin, Von Willebrand factor (vWF) and fibronectin (Lee et al., 2001). It is involved in hemostasis, thrombosis and inflammation events. CD36 is an adhesion molecule for monocytes, platelets, and ECs. Initially it was though that the expression of CD36 on erythroid progenitors declined with the maturation, but in the early 1990s van Schravendijk et al. showed that CD36 is also present on the surface of normal adult RBC. They also showed that its expression appears to be physiologically significant during infection with *P. falciparum*, when CD36 acts as a receptor for rosetting of *P. falciparum*-infected RBC with uninfected RBC: in fact, CD36 is defined as a scavenger receptor that bind numerous ligands, including the selective interaction with a specific domain of the *P. falciparum* erythrocyte membrane protein1 (PfEMP1) (Glenister et al., 2009; Cabrera et al., 2019). Moreover, low expression of this receptor may be sufficient for important and physiological interactions leading to cell adhesion, not only in malaria (Handunnetti et al., 1992). A high expression of CD36 was discovered in SCD RBC, and plays a key role in the adhesion of sickle cells to the endothelium and the consequent vaso-occlusive process (Lee et al., 2001; Sakamoto et al., 2013). Lastly, it is also shown that trauma-hemorrhagic shock induces an increase of several RBC adhesion molecules including CD36 which are responsible for the adhesiviness to the endothelial receptors like integrin α_v_β_3_ and VCAM-1 and the development of microvascular dysfunction (Deitch et al., 2014).

### Cluster of Differentiation 47 (CD47)

Cluster of differentiation 47 is an integrin-associated transmembrane protein (IAP) that has high affinity for thrombospondin (TSP) and signal-regulatory protein alpha (SIRPα) on the macrophage membrane (Lutz, 2004). CD47 is directly involved in the prevention of phagocytosis by macrophages through interaction with SIRPα on the macrophage surface inducing downstream inhibitory signaling (Klei et al., 2017). RBC that lack CD47 or express CD47 with switched conformation, are rapidly cleared from the circulation by splenic red pulp macrophages. In case of oxidative stress or aging, CD47 undergoes a conformational change, which triggers TSP binding and this altered/oxidized additionally is a recognition signal for SIRPα that indicates an intracellular damage (Burger et al., 2012). Moreover, Brittain et al. showed that CD47 mediates the adhesion of sickle RBC to immobilized TSP under both flow and static conditions. This leads to the adhesion of the RBC to the blood vessel wall contributing to vaso-occlusive crises in SCD (Brittain et al., 2001). CD47 could also play a very important and fundamental role in prevention of storage lesion of blood products and the early removal of RBC after transfusion. It has been suggested that reduction of CD47 expression on RBC, as a result of senescence or storage, is due to oxidative stress (Burger et al., 2012). It has also been shown that, since CD47 and Rh proteins are expressed as a complex on the RBC surface, in Rh_null_ individuals the expression of CD47 is less than 25% of normal levels. Consequently, those individuals show hemolytic anemia, reticulocytosis, and stomatocytosis that can be corrected by splenectomy (Oldenborg et al., 2000).

### Cluster of Differentiation 147 (CD147)

Cluster of differentiation 147 is part of the immunoglobulin superfamily, highly glycosylated and associated with GPA into the band 3 complex. This protein is expressed in mature RBC as a carrier molecule for the blood group antigen Ok system (also known as BASIGIN or EMMPRIN) (Coste et al., 2001). It is also considered as an adhesion molecule in multiple circumstances: for example it is known to be the direct receptor for PfRh5, which is a parasite ligand essential for *P. falciparum* blood stage growth (Crosnier et al., 2011). It was also demonstrated that CD147 is involved in promoting *P. falciparum* parasite invasion into RBC: this invasion can be inhibited by using the humanized monoclonal antibody HP6H8 against CD147 that blocks the interaction with a specific rhoptry-associated protein (RAP2) on the merozoites surface (Crosnier et al., 2011; Zenonos et al., 2015; Muramatsu, 2016; Zhang et al., 2018). Moreover, the group of Coste showed that CD147 plays a critical role in the re-entry of mature RBC from the spleen into the general circulation and when CD147 presence on the membrane was masked by antibody in mice, the migration out of the spleen was blocked and the RBC selectively trapped, inducing anemia and de novo erythropoiesis (Coste et al., 2001).

### Complement Inhibitors CD55 and CD59, and Cellular Adhesion Molecule CD44

Three other glycoproteins that have shown to confer an important role are CD55, or decay accelerating factor (DAF), CD59, and CD44. The first two are complement inhibitors anchored to the RBC membrane by glycosylphosphatidylinositol (GPI), and responsible for the regulation of the autologous lysis system. CD55 binds the complement 3 (C3) convertase, limiting the formation of complement 5 (C5) convertase and formation of the membrane attack complex. In this way this glycoprotein protects RBC against lysis mediated by natural killers cells, and also functions also as ICAM and as a receptor for viruses, like Echoviruses and coxsackie B viruses, and microorganisms. For example, it was shown that CD55-null RBC are resistant to invasion by *P. falciparum* due to failure to attach properly to the RBC surface (Lelliott et al., 2015; Egan, 2018). In fact, polymorphisms in CD55 are more prevalent in populations endemic for malaria infection, indicating a possible selection pressure on this gene (Egan et al., 2015).

CD59 is a major inhibitor of the terminal complement pathway. It blocks complement 8 (C8) and complement 9 (C9) in the assembling membrane attack complex for the pore formation (Richaud-Patin et al., 2003). Defects in the biosynthesis of GPI causes paroxysmal nocturnal hemoglobinuria (PNH), a hematological disease characterized by increased intravascular hemolysis and complement activation due to the absence of CD55 and CD59 (Alegretti et al., 2012; Brodsky, 2015). In systemic lupus erythematosus patients, hematologic abnormalities are common and the expression of CD55 and CD59 is decreased (Alegretti et al., 2010).

CD44 is a single pass transmembrane glycoprotein involved in cell-cell communication. It is the receptor for hyaluronic acid (HA), osteopontin, and fibronectin (Telen, 2005; Xu, 2011): in fact, its glycosylation affects its affinity to HA (Aoki, 2017). Also CD44 is a host factor required for efficient invasion of RBC by *P. falciparum* (Lelliott et al., 2015).

### Rh/RhAG Complex

The Rh blood group system is a complex association of membrane polypeptides composed of non-glycosylated Rh proteins and RhAG (Avent and Reid, 2000). In RBC, Rh is configured as a tetramer of two Rh and two RhAG subunits. Rh family proteins have an important clinical role in transfusion medicine due to their strong antigenic properties. The Rh complex contributes to the membrane stability and structure of RBC. In normal healthy conditions, Rh proteins are involved in the transport of NH_4_^+^ (Nakhoul and Hamm, 2004) and they are responsible, together with aquaporin-1, for half of normal CO_2_ permeability (Endeward et al., 2008). In fact, RhAG possesses a gas channel for the passage of CO_2_ in addition to NH_3_ (Endeward et al., 2008). The Rh null phenotype is described as an inherited condition in which various Rh antigens deficiencies result in a clinical syndrome characterized by a hemolytic anemia of varying severity (Ripoche et al., 2004).

## Plasma Proteins Mediated Interactions

### Thrombospondin (TSP)

Thrombospondin can be found as an immobilized extracellular matrix protein as well as a soluble plasma protein. It can facilitate adherence as a bridging molecule between RBC and ECs or platelets (Telen, 2005). Following concomitant expression of CD36 on both RBC and ECs, TSP can form a connection between these receptors (Trinh-Trang-Tan et al., 2010). In addition, TSP can interact with PS on RBC through the heparin-binding domain of CD36 (Betal and Setty, 2008). Other potential TSP receptors present on the RBC are CD47 and sulfated glycolipids. Conversely, TPS binds to α_v_β_3_ integrin expressed on the EC surface (Manodori et al., 2000; Brittain et al., 2001; Setty et al., 2002; El Nemer et al., 2007; Kaul, 2008). It has been demonstrated that, upon addition of heparin, TSP binding can be inhibited (Gupta et al., 1999). Soluble TSP also induces an activation pathway via binding to RBC CD47, which in turn activates α_4_β_1_ for extra adhesion to immobilized TSP (Brittain et al., 2004; Heppel, 2008). Activation of this signaling pathway is enhanced when RBC are exposed to shear stress (Telen, 2005). Concerning the interaction with platelets, TSP could also be a bridging molecule between RBC and platelets since it is known that TSP can also bind CD47, CD36, α_4_β_1_, PS, and sulfated glycolipids on RBC (Betal and Setty, 2008; Heppel, 2008; Trinh-Trang-Tan et al., 2010). The CD36-TSP interaction activates the CD47-dependent pathway. CD47 can bind to platelet α_IIb_β_3_ integrin, which in turn can bind ICAM-4 (Lagadec et al., 2003). TSP expression was found markedly increased in the vessel wall in multiple diseases like cardiovascular disorders, diabetes mellitus, atherosclerosis and ischemia-reperfusion injury (IRI; (Csányi et al., 2012).

### Von Willebrand Factor (vWF)

Von Willebrand factor serves as a bridging molecule between the endothelial receptor α_v_β_3_ and/or the endothelial receptor glycoprotein Ib, to a yet-unknown receptor on the RBC in a platelet-independent way (Setty et al., 2002; Smeets M. et al., 2017). Adhesion mediated by vWF seems to have a quantitatively and qualitatively different role in large vessel endothelium compared to the microvascular endothelium (Brittain et al., 1992). In general, increased RBC stress is linked to the pathology of several diseases including SCD, sepsis, chronic kidney disease, hemolytic uremic syndrome, hepatic failure, Wilson’s disease, diabetes, Alzheimer’s disease, and thrombotic thrombocytopenic. In all these conditions, RBC binds to ECs through interaction with monomer vWF and long multimers vWF, organized in large insoluble fibers. This leads to microangiopathic vascular damage, impairing the blood flow with the subsequent (multiple) organ failure (Smeets M.W. et al., 2017). The adhesion molecule that has been shown to interact with vWF and therefore promotes binding to RBC is PS (Nicolay et al., 2018). This interaction exists due to Annexin V molecule, which cannot only bind to PS on the RBC surface but also to vWF and thereby anchor the PS-exposing membrane to vWF or to vessel wall (Nicolay et al., 2018). Another study demonstrated that under reduced vascular wall shear stress, RBC bind specifically to vWF, forming the aggregates structure of venous thrombi (Smeets M. et al., 2017). Upon increased levels of intracellular Ca^2+^, which can occur in sickle cells but also normal RBC (Bogdanova et al., 2013), RBC can adhere to vWF strings which are connected to the endothelium by P-selectin or α_v_β_3_ integrin on the luminal surface (Smeets M.W. et al., 2017) and also by the endothelial glycocalyx (Kalagara et al., 2018). The group of Sultana et al. showed that sickle cells that are incubated with ECs in presence of multimers of vWF resulted in an increase of ICAM-1, E-selectin and VCAM-1 expression in the ECs, which in turn facilitated the adhesion of the sickle cells to the ECs (Sultana et al., 1998).

### Laminin Alpha 5

Laminin is an extracellular matrix glycoprotein also found in the sub-endothelium (Hillery et al., 1996; Wautier and Wautier, 2013). It has been shown that laminin alpha 5 chain can bind to Basal-cell adhesion molecule/Lutheran blood group glycoprotein (B-CAM/LU) on the RBC membrane. B-CAM/LU, or CD239, plays a crucial role in vaso-occlusion in SCD (Wautier and Wautier, 2013). On normal RBC B-CAM/LU is a relatively inactive receptor of laminin alpha 5, but it is highly expresses on sickle RBC (de Oliveira and Saldanha, 2010). The interaction of B-CAM/LU with laminin alpha 5 is inhibited when interacting in cis conformation, with GPC-derived sialic acid residues on the RBC. Upon loss of this interaction during aging, B-CAM/LU can interact, in trans, with sialic acid on laminin alpha 5 (Klei et al., 2018). In polycythemia vera, a myeloproliferative disorder characterized by a high occurrence of thrombosis, there is a correlation between the mutation of the janus kinase and phosphorylation of B-CAM/LU, which in turn initiates the interaction with endothelial laminin alpha 5 (Wautier et al., 2007; Wautier and Wautier, 2013). B-CAM/LU is directly linked to the RBC cytoskeleton via spectrin (An et al., 2008). This adhesion process is under the influence of epinephrine that activates B-CAM/LU through the cAMP-PKA pathway (Hines et al., 2003; Maciaszek et al., 2013; Wautier and Wautier, 2013; Zhang et al., 2017). It seems that the adhesion of RBC to ECs mediated by B-CAM/LU depends on its phosphorylated status and not on the amount of expressed receptors (Chaar et al., 2014). Phosphorylation of B-CAM/LU weakens the interaction with spectrin, which is accompanied by enhanced cell adhesion to laminin (An et al., 2008). The weakening of B-CAM/LU-spectrin interaction enables these molecules to aggregate and generate a larger adhesive force (Maciaszek et al., 2013). Hydroxycarbamide decreases the phosphorylation of B-CAM/LU and consequently reduces cell adhesion (Bartolucci et al., 2010; Chaar et al., 2014). It can therefore be used as a therapy in SCD patients. Interestingly, when ICAM4-α_v_β_3_ interactions are abolished, the adhesive capacity of laminin strongly decreases, suggesting that laminin-mediated interactions might function as a secondary adhesive interaction after ICAM4 interacted with α_v_β_3_ integrin (Ko et al., 2005; Zennadi et al., 2008).

### Fibrinogen

Fibrinogen is a plasma glycoprotein. The concentration of fibrinogen can be elevated under pathological conditions, such as bleeding tendency, liver disease (Tsang et al., 1990). In cerebrovascular dysfunction, high levels of fibrinogen lead to inflammation with accumulation of plasma proteins and increased vascular permeability which promotes hypercoagulation and thrombogenesis (Muradashvili and Lominadze, 2013). It was shown that the increase of fibrinogen correlates with elevated levels of C-reactive protein (CRP), which has been connected to thrombotic events, and the erythrocyte sedimentation rate (ESR): these last two are used as diagnostic parameters (Bitik et al., 2015; Flormann et al., 2015). In addition, fibrinogen has been shown to be able to adhere to different receptors on RBC, such as CD47, and on platelets with α_IIb_β_3_–like integrin (Carvalho et al., 2010; De Oliveira et al., 2012). Fibrinogen can also bind to ICAM1 on platelets (Massberg et al., 1999). Lastly, it was observed that sickle deoxygenated RBC tend to stick together and this occurs in presence of fibrinogen (Weiss et al., 2011).

### Immunoglobulin G (IgG)

Immunoglobulin G is the most abundant and common type of antibody, representing 75% of all plasma antibodies (Painter, 1998). The opsonization of specific targets, in particular band 3 by IgG, is essential for recognition of senescent RBC by macrophages (Meinderts et al., 2017). IgG specifically co-localizes with membrane aggregates composed by band 3, partially denatured hemoglobin and complement factor C3 (Badior and Casey, 2017). The group of Janvier et al. described that warm IgG autoantibodies are specific for the third external loop of band 3 and this is the major target in patients with warm antibody autoimmune hemolytic anemia (AIHA) (Janvier et al., 2013). It has also been suggested that a correlation between the decrease in sialic acid content of senescent RBC and accumulation of autologous IgG on the membrane has also been suggested to play an important role in physiological erythrophagocytosis (Ensinck et al., 2006).

### Lactadherin (MFG-E8)

Lactadherin or milk fat globule-EGF factor 8 (MFG-E8) is a plasma protein bridging molecule that plays a role as a cell adhesion protein. It contains an EGF-like domain at the amino terminus with the RGD sequence, a motif of Arginine, Glycine, and Aspartate aminoacids, and two C-domains at the carboxy terminus (Raymond et al., 2009). This protein is known to participate in a wide variety of cellular interactions, including phagocytosis of apoptotic cells (de Back et al., 2014). Lactadherin promotes engulfment of PS-containing apoptotic cells by macrophages (Guchhait et al., 2007). It has been shown that the presence of lactadherin enhaces and mediates the adhesion of PS-exposing sickle RBC to the endothelium via the α_v_β_3_ integrin (Guchhait et al., 2007). Lastly, it was shown that activated ECs are able to phagocyte PS-exposing and rigid RBC under both static and flow conditions: this mechanism could lead to ECs loss and contribute to vasopathological effects as in SCD (Fens et al., 2008, 2012).

### Growth Arrest Specific 6 (Gas6) and Protein S

Growth arrest specific 6, together with Protein S, is a ligand for tyrosin-protein kinase receptors like Tyro3, Axl and Mer (TAM): these are essential for the efficient phagocytosis of apoptotic cells. The TAM signaling appears to be autocrine/paracrine: since the TAM positive cells also produce the ligands (Lemke, 2013). This ligand physically links a TAM receptor expressed on the membrane of a phagocyte to PS-exposing cells suggesting that PS-exposing RBC are cleared via macrophages recognizing these PS/ligand binding (de Back et al., 2014).

## RBC-Derived Microparticles Mediated Interactions

Microparticles (MPs) derived from RBC are membranous extracellular structures shedded into the plasma under various circumstances. RBC-derived MPs size are 50–200 nm in diameter and they have an important role as key mediators of intercellular communication and consequently have an impact on various physiological processes such as blood homeostasis and modulation of immune responses (Dumaswala and Greenwalt, 1984; Willekens et al., 2008; Antonelou et al., 2010; Westerman and Porter, 2016). The reorganization of the membrane involving the lipid ratio and the disruption of the protein-protein interactions induce the microvesicles generation (Leal et al., 2018). MPs themselves expose antigens derived from the RBC membrane such as GPA, band 3, and PS on their membrane. PS can function to promote the coagulation cascade (Morel et al., 2011). By unknown mechanisms, RBC MPs contain a number of selected membrane components. For example, it was shown that MPs derived from old stored (20 days) RBC are rich in band 3, stomatin and PS (Lutz and Bogdanova, 2013). RBC-derived MPs levels are commonly elevated in *ex vivo* stored transfusion blood and during the course of some pathological conditions such as malaria, SCD, (Barteneva et al., 2013) and in hemolytic anemias (Litvinov and Weisel, 2017). Due to molecular defects, in hereditary membranophaties such as hereditary spherocytosis (HS) the instability of RBC membrane correlates with the membrane loss through vesiculation, with the outcome of less deformable RBC (Alaarg et al., 2013). Surprisingly, whereas HS RBC become more rigid, the extracellular vesicles in HS have a lower bending modulus (Vorselen et al., 2018). The role of RBC-derived MPs in different pathologies and mechanisms, such as inflammation, thrombosis and autoimmune reactions, is currently under investigation (Leal et al., 2018). The biological effects of RBC MPs comprise, for example, the regulation of coagulation mediated by binding to protein S (Koshiar et al., 2014), immune modulation, enhanced endothelial adhesion and scavenging action on nitric oxide (NO) (Said et al., 2018). RBC MPs especially appear to play a relevant role in the pathophysiology of SCD, promoting pro-inflammatory cytokine secretion, oxidative stress, endothelial apoptosis, leading to SCD vaso-occlusive crises (Said et al., 2018). The characterization of the MPs composition in specific blends of transmembrane proteins and lipids could lead to better understanding of interactions between specific cells or tissues.

## Dynamic Interactions

The motile nature of RBC has elicited multiple studies that focused on interactions from a spatial, mechanical and fluidic point of view. These interactions focus on rheologic dynamic, based on the nonspecific forces instead of the mediation by adhesion molecules (Lowe, 1988; Cabel et al., 1997).

### RBC Aggregation

Red blood cells are the most predominant blood cells and have a significant contribution to the fluidity of blood under physiological conditions (Simmonds et al., 2013). In fact, their ability to deform and aggregate contribute to blood viscosity high shear rates and low shear rates, respectively. RBC have the intrinsic tendency to form aggregates: it is possible to assist to this reversible phenomenon in which RBC assume the rouleau conformation, a specialy shaped structure composed by a linear arrays of stocked RBC, or with the 3D aggregates stasis (Baskurt and Meiselman, 2008). This mechanism occurs in health, aging and disease conditions and can be explained by two theories: the first is the bridging model, where the intercellular interaction is mediated by a protein or polymer such as fibrinogen or immunoglobulins. The second one is the depletion model, the most supported one, where protein or polymers are less concentrated near the RBC surface, creating an osmotic gradient (Wagner et al., 2013). According to the Fahraeus-Lindqvist effect, blood viscosity decreases with decreasing vessel diameters. In this way RBC migrate to the center of the vessel, leaving the plasma concentrated at the vessel wall. In various disease conditions, RBC aggregates increase blood viscosity and hydrodinamic resistence in large vessels, thereby promoting venus thrombosis (Weisel and Litvinov, 2019). Changes in plasma composition, such as during inflammatory reactions where fibrinogen level may rise fivefold, or increases in hematocrit are triggers that lead to serious hyperviscosity, intense aggregation and hydrodinamic clusters of RBC (Brust et al., 2014). This event is especially enhanced or abnormal in infections (sepsis), circulatory disorders (myocardial infarction), acute phase response, metabolic disordes, hematological disorders (polycythemia vera, SCD) and malignant diseases (Simmonds et al., 2013).

### RBC and ECs

The adhesion of RBC to other cell types and surfaces has been of particular interest due to the fact that this unusual aggregation and adhesiveness to other RBC and to ECs have been linked to various vascular disorders, such as SCD, DM, and hypertension (Setty et al., 2002; Wautier and Wautier, 2004). It was recently shown that non-absorbing macromolecules can have a marked impact on mediating the adhesion efficiency of RBC to ECs in patients with type 2 diabetes mellitus (T2DM) (Kaliyaperumal et al., 2019). Studies of nonspecific forces, like attractive, repulsive electrostatic forces, have considered macromolecular depletion as an effective mechanism inducing cell-surface adhesion (Neu and Meiselman, 2006). Other flow studies found different behavior of blood flow at microvascular level compared to macrovascular vessels due to the additional resistence of the endothelial glycocalyx layer on the luminal surface of the smaller blood vessel wall (Liu and Yang, 2009). This dynamic interaction of RBC and ECs involves the status of their glycocalyx located on the surfaces of both RBC and ECs. These layers facilitate “cushion” functions because the anionic properties of endothelial glycocalyx repels the negatively charged RBC, avoiding cells from coming to close (Liu and Yang, 2009). Endothelial glycocalyx can get damaged in metabolic syndrome, inflammatory processes and excess sodium intake (Noble et al., 2008; Lipowsky et al., 2011; Oberleithner et al., 2011). RBC glycocalyx get damaged due to increased oxidative stress as well as aging of the RBC (Neu et al., 2003). It seems that damaged EC glycocalyx leads to shedding of RBC glycocalyx and vice versa after these cells dynamically interact (Oberleithner, 2013). When a defect has occurred in either the glycocalyx of the RBC or the EC, this defect further enhances itself through the interaction of these cells with one another. Since the function of glycocalyx is prevention of adhesion (Liu and Yang, 2009), it could be that adhesiveness of RBC to ECs could increase when such a defect occurs. However, this has not yet been proven *in vivo*.

### RBC and Platelets

A different type of interplay that has been described between cells are hydrodynamic interactions. Platelets exhibit a phenomenon called platelet margination or lateral drift, in which the concentration of platelets is highest near the vessel wall (Namdee et al., 2013; Vahidkhahb et al., 2014). This seems to be the result of an interaction with RBC (Vahidkhah et al., 2013; Carboni, 2017). When a platelet comes across a RBC, either a crossing or a turning interaction occurs. In the crossing type of interaction the platelet rolls over the RBC, slightly deforming the flexible RBC, and moves in the same direction. In the turning type the platelet approaches the RBC, but then reverses back into the direction it came from without making direct contact with RBC. Whether crossing or turning occurs depends upon the initial lateral separation of the platelet and the RBC (Vahidkhah et al., 2013). When platelet-RBC interaction occurs in midflow of the vessel, this lateral separation is the only factor (Decuzzi et al., 2005). However, if the interaction occurs closer to the vessel wall, this affects the movement (Eckstein et al., 1988; Bächer et al., 2018). When a platelet is located farther away from the vessel wall than the RBC it is more likely to undergo a turning interaction trajectory, while when a platelet is located nearer to the vessel wall than the RBC crossing interaction is more likely (Vahidkhah et al., 2013). These events cause the platelet to be continuously driven away from the RBC-rich region in the center of the vessel into the plasma layer close to the vessel wall (Vahidkhah et al., 2013). Tokarev et al. suggested in their model that shear rate, hematocrit level and RBC size influence the mechanism in which the platelets are pushed against the vessel wall as a result of rebounding collision with an outrunning RBC or other blood cells (Tokarev et al., 2011; Fitzgibbon et al., 2015). These mechanisms could also explain why elevated hematocrit enhances platelet accumulation and binding to the vascular wall. It is also thought that an elevated hematocrit enlarges the interaction frequency of platelets with thrombi, which in turn accelerates the accumulation of platelets and thus the formation of thrombi (Yazdani and Em Karniadakis, 2016; Walton et al., 2017). Rigidity of RBC also plays a role: more rigid RBC increase platelet marginalization which increases sustainability for thrombosis (Aarts et al., 1986). In T2DM RBC and platelets exhibit abnormal biomechanical properties and biorheology which can affect blood flow dynamics and blood cell transport. In fact, less deformable T2DM RBC reduce the heterogenous collisions with the highest near-wall accumulation of T2DM platelets (Chang et al., 2018).

### RBC and White Blood Cells (WBC)

In order to roll on the endothelium, circulating WBC must migrate radially to contact the vessel wall (Munn and Dupin, 2008). This phenomenon is called margination and it is attributed to the RBC’s ability to aggregate and exclude WBC from the bulk solution. Munn and Dupin showed that rouleau formation of RBC is more effective in pushing WBC to the vessel wall than a loosely associated group of cells. WBC margination also depends on the local hematocrit, flow rate, RBC and WBC deformability (Fedosov and Gompper, 2014). RBC bounce the WBC against the endothelium and especially in small vessels WBC reverse the Fahraeus-Lindqvist effect and the resistant becomes greater due to the large size of the WBC (Munn and Dupin, 2008). In particular circumstances such as SCD, adherent WBC bind RBC and contribute to the microvascular pathology (Finnegan et al., 2007).

### RBC and Macrophages

The interaction between RBC and macrophages has been frequently discussed. Macrophages are directly involved in the two most delicate processes of RBC: erythropoiesis and erythrophagocytosis. So on one side macrophages are important for providing signals that induce differentiation and proliferation of RBC progenitors in the bone marrow niche. On the other side defective RBC are filtered, repaired and/or ultimately removed from the circulation by splenic and liver macrophages (Klei et al., 2017). Regarding these two fundamental stages specific and detailed reviews in health (de Back et al., 2014; Klei et al., 2017; Badior and Casey, 2018) and diseases (Mohanty et al., 2014; Risso et al., 2014; Flegel, 2015) are suggested. Since the focus of this review is on interactions that occur during RBC life, interactions involved in maturation and clearance are not discussed.

## RBC Interaction With Pathogens

Red blood cells do not only have the principal functions associated to oxygen and carbon dioxide transport and the role they play in homeostasis and blood flow distribution (Morera and MacKenzie, 2011), but RBC are also involved in the innate immune response (Minasyan, 2018). In a dynamic compartment such in the bloodstream, the clearance of bacteria is performed by oxycytosis. This means that bacteria moving with the blood flow, become triboeletrically charged and consequently attracted to RBC (Minasyan, 2016). This contact causes the release of oxygen from oxyhemoglobyn to the surface of the RBC and thereby killing the bacteria. This dimishes the triboelectric charge and the bacteria are finally washed from the RBC surface and digested in the liver or spleen (Minasyan, 2018). Although free hemoglobin may confer antimicrobial protection under homeostatic conditions, it has the opposite effect during disease, such as in severe sepsis often leading to increased mortality (Anderson et al., 2018). Many pathogens are able to bind glycophorins, such as reovirus, influenza C, sendai, mycoplasma pneumonie, *Escherichia coli* and ureaplasma urealyticum, acting as a chaperone and thereby avoiding the important tissues and facilitating the clearance of this pathogens by the spleen with macrophages (Baum et al., 2002). On the other hand it is via the same glycophorins that *P. falciparum* parasites adhere and invade the RBC (Morera and MacKenzie, 2011; Anderson et al., 2018). The malaria parasites multiply and evolve inside the RBC and are able to digest the hemoglobin converting it in crystals known as hemozoin (Parroche et al., 2007). This also happens for the HIV-1 virions and Zika virus in which they respectivly enrich the viral infectivity by binding to RBC (Beck et al., 2009) and hiding inside the RBC (Stone et al., 2017).

Thus, pathogens binding to or internalized into RBC could be both advantageous or detrimental to the host (Anderson et al., 2018).

## Conclusion

An overview of current knowledge on the interaction of RBC with other cells, ECs and platelets, in physiological and disease conditions, is presented here. Both direct interactions through receptors on the RBC and other key players, such as ECs, platelets, WBC, macrophages, other RBC, have been discussed, as well as indirect interactions between these cells (Supplementary Table S1). Indirect interaction can occur through plasma ligands, proteins and released molecules or particles from these cells. Other indirect interactions described in this review are mechanical: these kind of interactions are focused on the dynamic and rheological distribution of RBC in contact with other cells in physiological flow conditions. This underlines the complexity of the global interactions in which the mature RBC are involved and, more importantly, addresses a crucial attention to the pathological circumstances. Regarding the interaction with endothelium, depletion interaction plays an important role, while in interaction with platelets and WBC the margination caused by RBC is the largest contributing factor to interaction. Finally, we also reviewed the critical interaction of RBC with pathogens.

We note that studies on RBC interactions with other cells are, in most cases, conducted under artificial conditions that may differ from physiological conditions. For example, interaction with cultured ECs could be different compared to *in vivo* vascular ECs since cultured cells do not express the same structure and amount of glycocalyx *in vitro* as *in vivo* (Weinbaum et al., 2007). Also, often cells that are present *in vivo* are excluded in experimental setups to not influence the measurements. Finally, *in vitro* studies that are conducted, often use flat cultured monolayer of ECs, so the influence of the three-dimensional vessel wall is not taken into account. The largest amount of studies conducted on adhesiveness of RBC is performed using blood of SCD patients or rodents. These cells are active and express PS and other receptors on their membranes which enable them to adhere to other cells. However, it is not known if sickle cells adhere in the same manner as other activated RBC would do. With regard to receptor-based interactions there are probably multiple receptors and interactions responsible for adhesion of RBC. It is also not necessarily so that high-affinity mechanisms shown in laboratory studies are the largest contribution factor to adhesion *in vivo*. Microvascular blood flow can be intermittent and RBC can be slowed by passing granulocytes because of their larger size. In such conditions lower affinity mechanism may also play an important role in adhesion.

It is recurrently observed that in different pathological conditions, such as SCD, T2DM, G6PD deficiency, thalassemia, chronic diseases, malaria, RBC become more adhesive and less deformable and this enhances the possible interactions with the surrounding cells. This leads to the development of microvascular obstructions with consequential impaired oxygen and nutrient delivery to organs and tissues which can cause organ failure.

To conclude, this overview goes beyond the characteristics of the RBC itself to yet expand our knowledge concerning RBC function and crucial roles. Studying the interactions between RBC and other cell types, proteins, pathogens or other cellular mediators will therefore increase our understanding of RBC homeostasis and contribute to novel insights in a variety of hematological and non-hematological disorders.

## Author Contributions

All authors listed have made a substantial, direct and intellectual contribution to the work, and approved it for publication.

## Conflict of Interest Statement

The authors declare that the research was conducted in the absence of any commercial or financial relationships that could be construed as a potential conflict of interest.

## Supplementary Material

TABLE S1 Summary of the classified interactions with the key player factors and the pathologies involved. The key players of each specific interaction and pathologies correlated are connetted with the same corresponding number (ECs = endothelial cells; SCD = sickle cell disease; G6PD = glucose-6-phosphate dehydrogenase; WBC = white blood cells; PNH = paroxysmal nocturnal hemoglobinuria; MPs = microparticles).
