# Supplementary material for: Red Blood Cells: Chasing Interactions
Source: Front Physiol. 2019 Jul 31;10:945. doi: 10.3389/fphys.2019.00945 (PMC6684843; doi:10.3389/fphys.2019.00945)
Supplement: TABLE S1 — Summary of the classified interactions with the key player factors and the pathologies involved. The key players of each specific interaction and pathologies correlated are connetted with the same corresponding number (ECs = endothelial cells; SCD = sickle cell disease; G6PD = glucose-6-phosphate dehydrogenase; WBC = white blood cells; PNH = paroxysmal nocturnal hemoglobinuria; MPs = microparticles). [file Data_Sheet_1.PDF]

Factors	Interactions	Pathologies
**Transmembrane Transport-Proteins Mediated Interactions**		
1. Band3-protein<br>2. PIEZO1<br>3. KCNN4 (Gardos Channel)	1. Macrophages mediated by natural antibodies, ECs<br>2. Shear stress, mechanical forces<br>3. Indirectly-mediated by PIEZO1	1. Diabetes mellitus (risk of vascular complications)<br>2. Hereditary xerocytosis, Malaria<br>3. SCD (risk of vaso-occlusion), Hemolytic anemia, Gardos channelopathies
**Phospholipids Mediated Interactions**		
Phosphatidylserine	ECs, platelets, macrophages	SCD (risk of adhesion), G6PD deficiency, thalassemia, iron deficiency, diabetes mellitus, chronic diseases
**Glycoproteins Mediated Interactions**		
4. Glycophorins<br>5. ICAM-4<br>6. CD36<br>7. CD47<br>8. CD147<br>9. CD55 and CD59<br>10. CD44<br>11. Rh/RhAG complex	4. Pathogens<br>5. WBC, ECs, platelets mediated by integrins<br>6. Monocytes, platelets, ECs<br>7. Macrophages, ECs<br>8. Parasites<br>9. Viruses, microorganisms<br>10. Extracellular matrix, parasites<br>11. unknown	4. Malaria<br>5. SCD (risk of vaso-occlusion)<br>6. Malaria, SCD, microvascular dysfunctions<br>7. SCD<br>8. Malaria<br>9. PNH, lupus erythematosus<br>10. Malaria<br>11. Hemolytic anemia of varying severity
**Plasma Proteins Mediated Interactions (bridging molecules)**		
12. Thrombospondin<br>13. Von Willebrand Factor<br>14. Laminin alpha 5<br>15. Fibrinogen<br>16. IgG<br>17. Lactadherin<br>18. Gas6 and protein S	12. ECs, platelets<br>13. ECs<br>14. ECs<br>15. Platelets<br>16. Macrophages<br>17. Macrophages, ECs<br>18. Macrophages	12. Cardiovascular disorders, diabetes mellitus, atherosclerosis, ischemia-reperfusion injury<br>13. SCD, sepsis, chronic kidney, hemolytic uremic syndrome, hepatic failure, Wilson's disease, diabetes, Alzheimer's disease, thrombotic thrombocytopenic<br>14. SCD, polycythemia vera<br>15. Bleeding tendency, liver disease, cerebrovascular dysfunction<br>16. Warm antibody autoimmune hemolytic anemia<br>17. SCD (risk of vasopathological effects)<br>18. unknown
**RBC-derived Microparticles Mediated Interactions**		
MPs	ECs, macrophages	Malaria, SCD, hemolytic anemias, hereditary spherocytosis
